# Single-Cell RNA-Seq of Cisplatin-Treated Adult Stria Vascularis Identifies Cell Type-Specific Regulatory Networks and Novel Therapeutic Gene Targets

**DOI:** 10.3389/fnmol.2021.718241

**Published:** 2021-09-09

**Authors:** Ian A. Taukulis, Rafal T. Olszewski, Soumya Korrapati, Katharine A. Fernandez, Erich T. Boger, Tracy S. Fitzgerald, Robert J. Morell, Lisa L. Cunningham, Michael Hoa

**Affiliations:** ^1^Auditory Development and Restoration Program, National Institute on Deafness and Other Communication Disorders, National Institutes of Health, Bethesda, MD, United States; ^2^Laboratory of Hearing Biology and Therapeutics, National Institute on Deafness and Other Communication Disorders, National Institutes of Health, Bethesda, MD, United States; ^3^Genomics and Computational Biology Core, National Institute on Deafness and Other Communication Disorders, National Institutes of Health, Bethesda, MD, United States; ^4^Mouse Auditory Testing Core Facility, National Institutes of Health, Bethesda, MD, United States

**Keywords:** stria vascularis, endocochlear potential, inner ear, scRNA-Seq, cisplatin, differential expression, gene regulatory networks

## Abstract

The endocochlear potential (EP) generated by the stria vascularis (SV) is necessary for hair cell mechanotransduction in the mammalian cochlea. We sought to create a model of EP dysfunction for the purposes of transcriptional analysis and treatment testing. By administering a single dose of cisplatin, a commonly prescribed cancer treatment drug with ototoxic side effects, to the adult mouse, we acutely disrupt EP generation. By combining these data with single cell RNA-sequencing findings, we identify transcriptional changes induced by cisplatin exposure, and by extension transcriptional changes accompanying EP reduction, in the major cell types of the SV. We use these data to identify gene regulatory networks unique to cisplatin treated SV, as well as the differentially expressed and druggable gene targets within those networks. Our results reconstruct transcriptional responses that occur in gene expression on the cellular level while identifying possible targets for interventions not only in cisplatin ototoxicity but also in EP dysfunction.

## Introduction

The stria vascularis (SV) is a stratified epithelial tissue located in the lateral wall of the mammalian cochlea that generates and maintains the endocochlear potential (EP) and regulates the secretion of endolymph (Wangemann, 2002; Chang et al., 2015). Endolymph possesses a high potassium concentration that creates a positive potential of 80–120 mV in the endolymph-containing scala media relative to the perilymph-containing compartments of the cochlea. The EP is the driving force for hair cell mechanoelectrical transduction and hearing. SV cell types, which express several critical ion channels, work together to generate the EP. Importantly, mutations in SV ion channel genes related to EP generation result in hearing loss in humans (Wangemann, 2002).

More broadly, mutations in genes expressed by SV cell types result in hearing loss in mammals. In mouse, mutations in genes expressed by marginal cells of the SV, including *Slc12a2*, *Atp1b2*, *Kcnq1*, and *Kcne1*, result in a loss or reduction of EP and deafness (Delpire et al., 1999; Dixon et al., 1999; Wangemann, 2002; Chang et al., 2015; Faridi et al., 2019; McNeill et al., 2020; Mutai et al., 2020). *Slc12a2* encodes Na+/K+/Cl– symporter 1 (NKCC1), which is expressed at the basolateral membrane of SV marginal cells and is responsible for transporting potassium from the intrastrial space into marginal cells (Wangemann, 2002). The loss of SLC12A2 results in deafness in mouse models (Delpire et al., 1999; Dixon et al., 1999; Wangemann, 2002) and has recently been implicated in human hearing loss (McNeill et al., 2020; Mutai et al., 2020). *Atp1b2* encodes the β2 subunit of Na+, K+-ATPase (NKA) protein, which has been shown to be expressed in the basolateral membranes of mouse SV marginal cells (Konishi et al., 1978; Schulte and Steel, 1994; Wangemann et al., 1995; Wangemann, 2002). Similar to NKCC1, NKA pumps out cations from the SV to maintain a high potassium concentration and positive EP in the endolymph in the scala media (Nishiyama et al., 1994; Furukawa et al., 1996; Lang et al., 2007; Nin et al., 2008). ATP1A1 and ATP1B2, encoded by *Atp1b2*, form the Na+/K+-ATPase that is essential for maintaining the low potassium concentration in the intrastrial space and is important for the generation of the +80 mV endocochlear potential in the scala media (Wangemann, 2002). Decline in EP is associated with the loss of Na+/K+-ATPase activity in the stria vascularis (Gratton et al., 1996) and direct inhibition of the Na+/K+-ATPase with ouabain has been shown to reduce the EP (Nin et al., 2008). These data suggest that *Atp1b2* is essential for EP generation.

*Kcnq1* and *Kcne1* encode the voltage-gated potassium channel Kv7.1, which is expressed on the apical surface of the marginal cell and plays a crucial role in secreting potassium into the endolymph and maintaining the EP. Conditional *Kcnq1* null mice exhibit a collapse of Reissner’s membrane, loss of EP, and are deaf (Chang et al., 2015). Mutations in *KCNQ1*/*KCNE1* result in human deafness and have been reviewed previously (Faridi et al., 2019). Similarly, genes expressed by intermediate cells including *Kcnj10* and *Met* cause hearing loss in humans (Scholl et al., 2009; Reichold et al., 2010; Freudenthal et al., 2011; Mujtaba et al., 2015; Alabdullatif et al., 2017). Notably, *Kcnj10* encodes Kir4.1, which is an inwardly rectifying potassium channel expressed on the apical (marginal cell-facing) membranes of intermediate cells, that has been shown to be critical for EP generation with its loss resulting in hearing loss in both mice and humans (Marcus et al., 2002; Wangemann, 2002; Wangemann et al., 2004; Scholl et al., 2009; Reichold et al., 2010; Freudenthal et al., 2011; Chen and Zhao, 2014). Furthermore, loss of Ednrb has been shown to result in the reduction of EP (da-Eto et al., 2011). While loss of *Met* expression has not been demonstrated to specifically result in loss or reduction of EP, dysfunctional HGF/MET signaling resulting from a knock-in mouse of a human mutation of *HGF* has been implicated in the reduction of EP through a reduction in intermediate cells (Morell et al., 2020). Finally, mutations in genes expressed by SV basal cells, including *Cldn11*, *Gjb2* and *Gjb6* have been implicated in the reduction of EP and hearing loss. Loss of *Cldn11*, which encodes the CLDN11 protein that is expressed in tight junctions of SV basal cells, has been shown to result in deafness and reduced EP in *Cldn11* null mice (Gow et al., 2004; Kitajiri et al., 2004a). *Gjb2* and *Gjb6* encode gap junctions expressed by SV basal cells and, to a lesser extent, intermediate cells (Kikuchi et al., 1995; Xia et al., 1999, 2001). Mutations of *GJB2* and *GJB6* are associated with deafness in humans (Kelsell et al., 1997; Grifa et al., 1999) and have been shown to result in hearing loss and a reduction in EP in mouse (Teubner et al., 2003; Wingard and Zhao, 2015; Mei et al., 2017; Lin X. et al., 2019; Mammano, 2019; Wu et al., 2019). Thus, existing literature emphasizes the importance of genes and proteins expressed by SV cell types in both hearing and EP generation.

Previously, our group has utilized single-cell RNA-sequencing to characterize the gene regulatory networks of the major SV cell types, including marginal, intermediate, and basal cells, that underlie SV homeostatic functions, including EP generation in the adult mouse (Korrapati et al., 2019). Gene regulatory networks (GRNs) define and maintain cell type-specific transcriptional states, which in turn underlie cellular morphology and function (Aibar et al., 2017; Fiers et al., 2018). From this perspective, cells and their respective states are defined by a combination of transcription factors (TFs) that interact with a set of target genes to produce a specific gene expression profile. Perturbation of cell state through genetic, epigenetic, or pharmacologic means may provide insight into the contribution of these GRNs to cell function (Liberali et al., 2015; Li et al., 2018; Musa et al., 2018; Caldera et al., 2019). Diseases or pharmacologic treatments represent perturbations of GRNs and investigation of these states at the single cell level may shed light on critical GRNs that underlie human disease or responses to treatment by potentially identifying key regulators in these GRNs (del Sol et al., 2010; Fiers et al., 2018; Caldera et al., 2019).

Cisplatin is commonly used to treat patients with a variety of cancers and results in permanent hearing loss in 35–100% of treated adults due to its ototoxicity as reviewed by Paken et al. (2019). Recently, Breglio et al. (2017) demonstrated long term retention of cisplatin in the SV of both mice and humans, as measured by platinum concentration over time. These authors also demonstrated accompanying declines in EP in a mouse model of cisplatin ototoxicity that mimics the treatment and time course seen in humans undergoing cisplatin treatment for cancer (Breglio et al., 2017). In this model, cisplatin treatment results in temporary decline in the EP after the 1st cycle that gradually recovers but eventually becomes permanent after three cycles of cisplatin and accompanies hearing loss (Breglio et al., 2017; Fernandez et al., 2019). SV retention of cisplatin combined with the decline in EP and its known ototoxic effects in humans prompted us to investigate the underlying transcriptional changes in SV cell types associated with cisplatin exposure to identify GRNs involved in the loss of EP-generating function in the SV.

To investigate the acute transcriptional response of the SV to cisplatin instead of the cumulative effects of cisplatin exposure, a mouse model for acute cisplatin-induced ototoxicity consisting of the dose equivalent (14 mg/kg, IP) to one cycle of cisplatin was utilized based on the cisplatin-induced ototoxicity mouse model by Fernandez et al. (2019). Using this mouse model for acute cisplatin-induced ototoxicity to better understand the molecular mechanisms underlying EP generation and maintenance, we demonstrate that the decline in EP associated with cisplatin treatment is associated with changes in known SV cell type-specific EP-related genes at the protein and RNA levels by fluorescence immunohistochemistry and digital droplet qPCR (ddPCR), respectively.

To further investigate transcriptional changes associated with a decline in EP in response to cisplatin treatment, we performed single-cell RNA-sequencing (scRNA-Seq) of cisplatin-treated adult SV and compared these transcriptional profiles to our previously published unperturbed adult SV scRNA-Seq dataset (Korrapati et al., 2019). In addition to corroborating our observations with known EP-related genes, we identify cell type-specific gene regulatory networks associated with differentially expressed EP-related genes. Among the major SV cell types, we demonstrate that marginal and intermediate cells are differentially affected by cisplatin treatment and we define differentially expressed genes as they relate to select EP-related GRNs affected by cisplatin. Finally, we utilize Pharos^1^ to identify druggable gene targets in these GRNs that are preferentially affected by cisplatin. We pinpoint potentially repurposable FDA-approved drugs that may counteract transcriptional changes induced by cisplatin. More generally, analyses of these data serve as an example of a bioinformatic pipeline designed to identify druggable targets and potentially repurposable drugs to reverse not only cisplatin-induced hearing loss but also hearing loss related to stria vascularis dysfunction.

## Results

### Treatment With Cisplatin Results in a Reduction in EP but Without Obvious Structural Abnormalities

**FIGURE 1:**
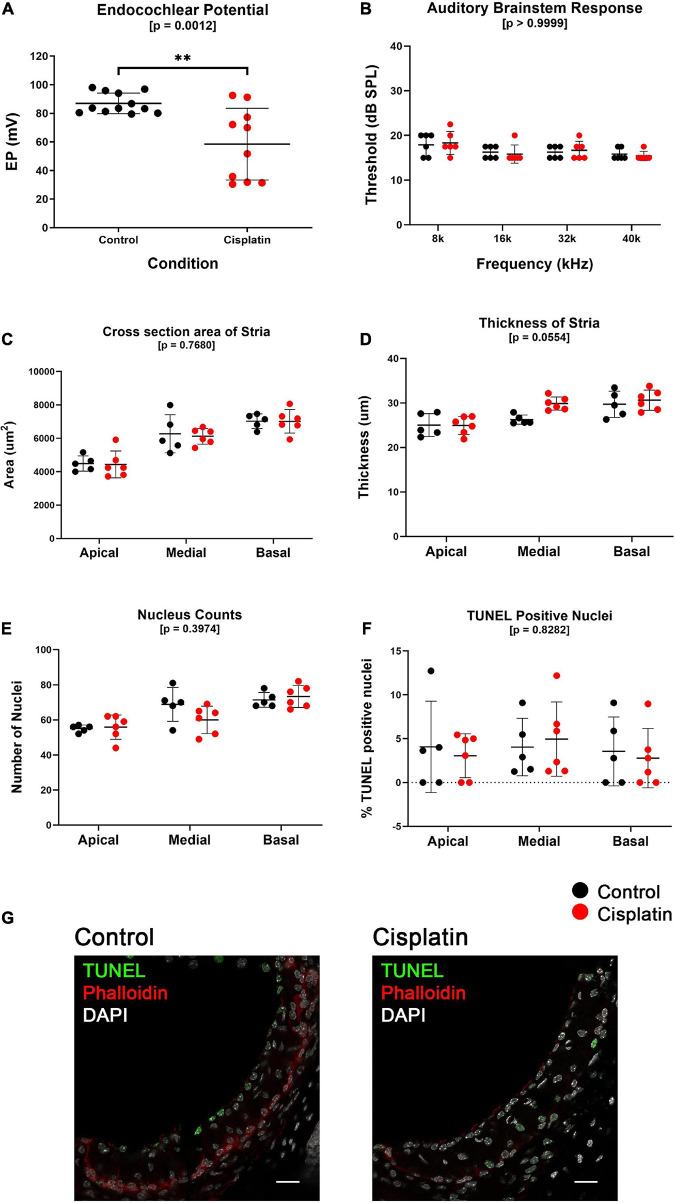
A single dose of cisplatin (14 mg/kg, IP) results in a reduction in EP after 24 h with no reduction in ABR threshold, minimal structural change, and no evidence of increased apoptosis. **(A)**, EP measurements obtained 24 h after treatment in control (*n* = 12) and cisplatin-treated (*n* = 10) demonstrate that cisplatin-treated mice have a reduced EP compared to control mice (*p* = 0.0012). **(B)** In a separate cohort of mice, ABR thresholds measured at the 8, 16, 32, and 40 kHz frequencies were unchanged between control (*n* = 6) and cisplatin-treated (*n* = 6) mice (two-way ANOVA, *p* > 0.9999). **(C)** SV cross-sectional area was unchanged between conditions in all cochlear turns (two-way ANOVA, *p* = 0.7680). **(D)** SV thickness was unchanged between conditions in all cochlear turns (two-way ANOVA, *p* = 0.0554). **(E)** Total nucleus counts in the SV were unchanged between conditions in all cochlear turns (two-way ANOVA, *p* = 0.3974). **(F)** TUNEL-positive cell nuclei, calculated as a percentage of the total nuclei, was unchanged between conditions in all cochlear turns (two-way ANOVA, *p* = 0.8282). **(G)** Representative TUNEL staining in SV cross-sections. For **(B–G)**, one cohort of mice were used, control (*n* = 6) and cisplatin-treated (*n* = 6). Scale bars: 20 μm. **p < 0.01.

Cisplatin-treated mice have a reduced EP compared to control mice (Control mean EP = 86.96 mV, Cisplatin-treated mean EP = 58.43 mV, *p* = 0.0012) (Figure 1A). EP measurements were obtained 24 h after treatment in control (*n* = 12) and cisplatin-treated (*n* = 10) mice that were given a single IP injection of saline or 14 mg/kg cisplatin, respectively. Given the constraints of performing auditory brainstem response (ABR) and EP recordings at the same time point, ABR thresholds (Figure 1B) and hair cell counts (Supplementary Figures S1A,B) were analyzed in a parallel cohort of control (*n* = 6) and cisplatin-treated (*n* = 6) mice. ABR thresholds measured at the 8, 16, 32, and 40 kHz frequencies were unchanged between control and cisplatin-treated mice (two-way ANOVA, *p* > 0.9999) (Figure 1B). Inner (two-way ANOVA, *p* = 0.6434) and outer (two-way ANOVA, *p* = 0.6423) hair cell counts between cisplatin-treated and control cochlea in the apical and basal turns were comparable and did not demonstrate a significant difference between conditions (Supplementary Figures S1A,B). DPOAE amplitudes between control and cisplatin-treated mice were unchanged in both left and right ears (Supplementary Figures S1C,D). Additionally, SV cross-sectional area in the apical, medial, and basal turns of the cochlea was unchanged between control and cisplatin-treated mice (two-way ANOVA, *p* = 0.7680) (Figure 1C). Similarly, SV thickness in the apical, medial, and basal turns of the cochlea was unchanged between control and cisplatin-treated mice (two-way ANOVA, *p* = 0.0554) (Figure 1D). Total nucleus counts in the SV in the apical, medial, and basal turns of the cochlea were unchanged between control and cisplatin-treated mice (two-way ANOVA, *p* = 0.3974) (Figure 1E). Furthermore, the number of TUNEL-positive cell nuclei, calculated as a percentage of the total nuclei, was unchanged between control and cisplatin-treated mice across apical, medial, and basal cochlear turns (two-way ANOVA, *p* = 0.8282) (Figure 1F). Representative TUNEL staining in SV cross-section co-labeled with DAPI and phalloidin are shown (Figure 1G). Together these data indicate that a single injection of 14 mg/kg cisplatin resulted in reduced EP and suggest that hearing loss and hair cell death at this time point is not significant.

### Expression of Key EP-Related Cell-Type Specific Proteins Is Reduced in Cisplatin-Treated Mice

**FIGURE 2:**
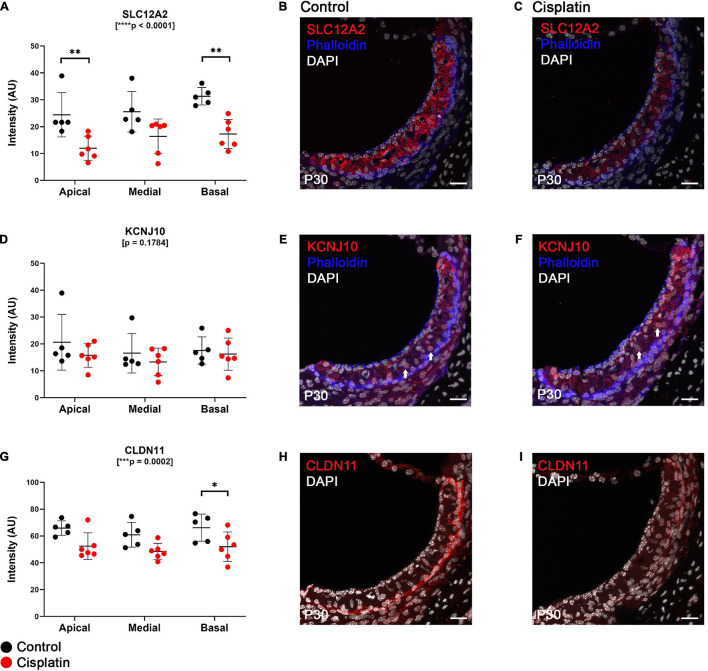
Expression of key EP-related cell-type specific proteins is reduced in the SV after cisplatin treatment. **(A)** Twenty four hours after cisplatin injection, SLC12A2 immunofluorescence intensity was significantly reduced in the apical and basal turns of the cochlea (two-way ANOVA, *p* < 0.0001; Sidak’s multiple comparisons test, *q* = 0.0068 and *q* = 0.0022, respectively). **(B,C)** Representative SLC12A2 immunostaining of SV cross sections of control and cisplatin-treated mice. **(D)** Immunofluorescence staining intensity of KCNJ10 was unchanged between control and cisplatin-treated mice (two-way ANOVA, *p* = 0.1784). **(E,F)** Representative KCNJ10 immunostaining of SV cross sections of control and cisplatin-treated mice. White arrows point to representative intermediate cell nuclei. **(G)** Immunofluorescence staining intensity of CLDN11 was reduced in the basal turn of cisplatin-treated compared to control mice (two-way ANOVA, *p* = 0.0002; Sidak’s multiple comparisons test *q* = 0.0418). **(H,I)** Representative CLDN11 immunostaining of SV cross sections of control and cisplatin-treated mice. Scale bars: 20 μm. *p < 0.05; **p < 0.01; ***p < 0.001; ****p < 0.0001.

SLC12A2 is expressed at the basolateral membrane of SV marginal cells, transports potassium from the intrastrial space into marginal cells and is critical for EP generation (Wangemann, 2002). We observed that at 24 h after cisplatin injection, SLC12A2 immunofluorescence intensity was significantly reduced in the apical and basal turns of the cochlea (two-way ANOVA, *p* < 0.0001; Sidak’s multiple comparisons test, *q* = 0.0068 and *q* = 0.0022, respectively) (Figure 2A), with representative cross sections of control (Figure 2B) and cisplatin-treated mice (Figure 2C). Immunofluorescence staining intensity of KCNJ10, which is known to be expressed on the apical (marginal cell-facing) membranes of intermediate cells (Marcus et al., 2002; Wangemann, 2002; Chen and Zhao, 2014), was unchanged between control and cisplatin-treated mice (two-way ANOVA, *p* = 0.1784) (Figure 2D), with representative cross sections of control (Figure 2E) and cisplatin-treated mice (Figure 2F). Finally, CLDN11 (claudin 11) immunofluorescence staining intensity, a tight junction protein expressed by SV basal cells, was reduced in the basal turn of cisplatin-treated compared to control mice (two-way ANOVA, *p* = 0.0002; Sidak’s multiple comparisons test *q* = 0.0418) (Figure 2G), with cross sections of control (Figure 2H) and cisplatin-treated mice (Figure 2I).

### ddPCR Demonstrates a Reduction in Several EP-Related Genes in Cisplatin-Treated SV Compared to Control

**FIGURE 3:**
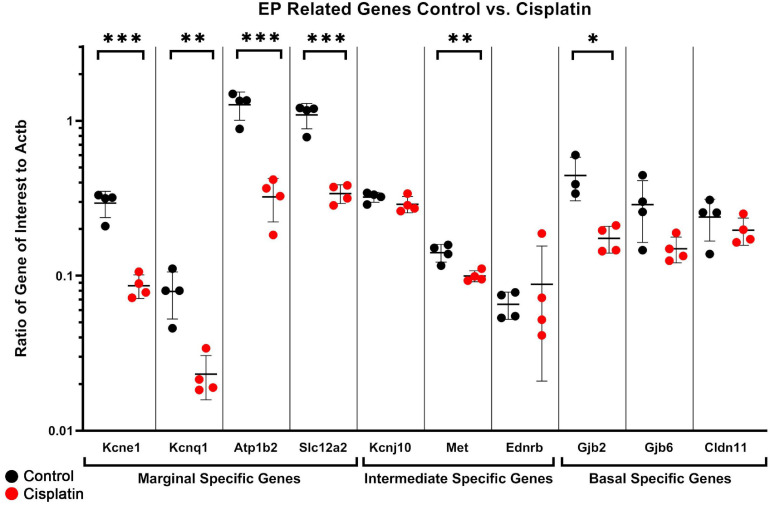
ddPCR of dissected SV demonstrates a reduction in several EP-related genes after cisplatin treatment. In whole dissected SV, we observed a significant reduction in selected EP-related genes specific to marginal cells: *Kcne1* (*q* = 0.0020), *Kcnq1* (*q* = 0.0161), *Atp1b2* (*q* = 0.0020), and *Slc12a2* (*q* = 0.0020) (unpaired multiple *t*-test, Benjamini–Hochberg correction). A significant reduction in two EP-related genes specific to intermediate cells: *Met* (uncorrected *p* = 0.0064) and *Kcnj13* (uncorrected *p* = 0.0117) was seen between control and cisplatin-treated mice (unpaired multiple *t*-test, Benjamini–Hochberg correction). Expression of other known intermediate cell-specific genes including *Kcnj10* (*p* = 0.1746) and *Ednrb* (*p* = 0.5309) were not reduced in cisplatin-treated compared to control. Expression of EP-related genes in basal cells, including *Gjb2* (*q* = 0.0207) was significantly reduced, while *Gjb6* (*q* = 0.1086), and *Cldn11* (*q* = 0.4012), were not decreased in cisplatin-treated compared to control mice (unpaired multiple *t*-test, Benjamini–Hochberg correction). *p < 0.05; **p < 0.01; ***p < 0.001.

As we have described, a number of EP-related genes have been identified including *Kcne1*, *Kcnq1*, *Atp1b2*, *Slc12a2*, *Kcnj10*, *Met*, *Cldn11*, *Gjb2*, and *Gjb6* (Wangemann, 2002; Gow et al., 2004; Kitajiri et al., 2004b; Morell et al., 2020). We utilized ddPCR of whole isolated SV to quantify the effect of cisplatin-treatment on the expression of these EP-related genes in the SV to gain a better sense of the transcriptional response with respect to EP-generating mechanisms. In whole isolated SV, we observed a significant reduction in selected EP-related genes specific to marginal cells: *Kcne1* (*q* = 0.0020), *Kcnq1* (*q* = 0.0161), *Atp1b2* (*q* = 0.0020), and *Slc12a2* (*q* = 0.0020) (unpaired multiple *t*-test, Benjamini–Hochberg correction) (Figure 3). A significant reduction in an EP-related gene specific to intermediate cells: *Met* (uncorrected *p* = 0.0064) was seen between control and cisplatin-treated mice (unpaired multiple *t*-test, Benjamini–Hochberg correction) (Figure 3). Expression in whole dissected SV of other known EP-related intermediate cell-specific genes including *Kcnj10* (*p* = 0.1746) and *Ednrb* (*p* = 0.5309) were not reduced in cisplatin-treated compared to control. *Ednrb* expression has previously been identified in the stria vascularis (Matsushima et al., 2002; Stanchina et al., 2006; Xu et al., 2010; da-Eto et al., 2011) and is identified as an intermediate cell-specific gene in the dataset published by Korrapati et al. (2019) as shown in the gene Expression Analysis Resource (gEAR)^2^. Finally, expression of EP-related genes in basal cells, including *Gjb2* (*q* = 0.0207) was significantly reduced, while *Gjb6* (*q* = 0.1086), and *Cldn11* (*q* = 0.4012), were not decreased in cisplatin-treated compared to control mice (unpaired multiple *t*-test, Benjamini–Hochberg correction) (Figure 3). Both GJB2 and GJB6 protein have been previously shown to be expressed by basal cells and intermediate cells (Lang et al., 2007; Wangemann et al., 2007; Nickel and Forge, 2008; Liu W. et al., 2009; Locher et al., 2015; Liu W. et al., 2016). CLDN11 is a well-established specific marker for basal cells in the stria vascularis (Gow et al., 2004; Kitajiri et al., 2004a; Liu W. et al., 2017; Korrapati et al., 2019). The limited sensitivity of ddPCR to changes at the cell type level when assaying whole SV tissue may account for the inability to detect changes in cell type-specific genes between control and cisplatin-treated mice. Nonetheless, ddPCR analysis of cisplatin-treated SV suggest that RNA transcription related to EP generation is adversely affected by cisplatin. Due to our limited ability to resolve cell type-specific responses in the SV in response to cisplatin and to gain a more comprehensive understanding of the transcriptional response to cisplatin at the cellular level in the SV, we then utilized scRNA-Seq to perform transcriptional profiling of the SV in cisplatin-treated adult mice.

### scRNA-Seq of Cisplatin-Treated Adult SV Demonstrates Similar Cell Populations as scRNA-Seq of Untreated Adult SV

**FIGURE 4:**
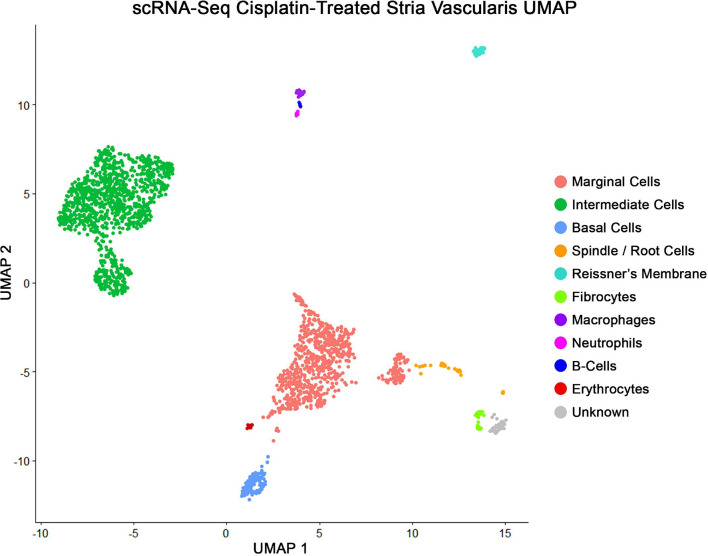
scRNA-Seq of cisplatin-treated adult SV demonstrates all the expected cell populations as untreated adult SV. Uniform manifold and projection (UMAP) clusters identified using published markers, showing marginal, intermediate, and basal cells in pink, green, and blue, respectively. Each dot represents a single whole cell. Additional cell types identified were spindle cells, fibrocytes, Reissner’s membrane cells, neutrophils, B-cells, macrophages, and red blood cells (RBCs).

Major cell types in cisplatin-treated SV were identified using published markers (Korrapati et al., 2019), demonstrating the capture of marginal, intermediate, and basal cells in pink, green, and blue, respectively, using a uniform manifold and projection (UMAP) clustering (Figure 4). The genes *Kcne1* and *Kcnq1* were used as markers to identity marginal cells, *Kcnj10* and *Cd44* were used to identify intermediate cells, and *Cldn11* and *Tjp1* were used to identify basal cells. Additional clusters were identified using published markers and included cell types adjacent to and within the structure of the stria. These included the fibrocytes of the lateral wall, cells of Reissner’s membrane that separate the scala media from the scala vestibuli, the spindle cells at the edges of the SV, and various lymphoid, myeloid, and erythroid populations including neutrophils, B-cells, macrophages, and red blood cells (RBCs) (Figure 4). Bioinformatic clustering revealed similar SV cell type-specific clusters across both cisplatin-treated and untreated SV single cell RNA-Seq datasets (Korrapati et al., 2019). Cell type-specific gene expression in both the cisplatin-treated and control SV datasets is provided as a supplement (Supplementary Table S1).

### Transcriptional Changes in Response to Cisplatin Reveal a Preferential Effect on Marginal and Intermediate Cells in SV

**FIGURE 5:**
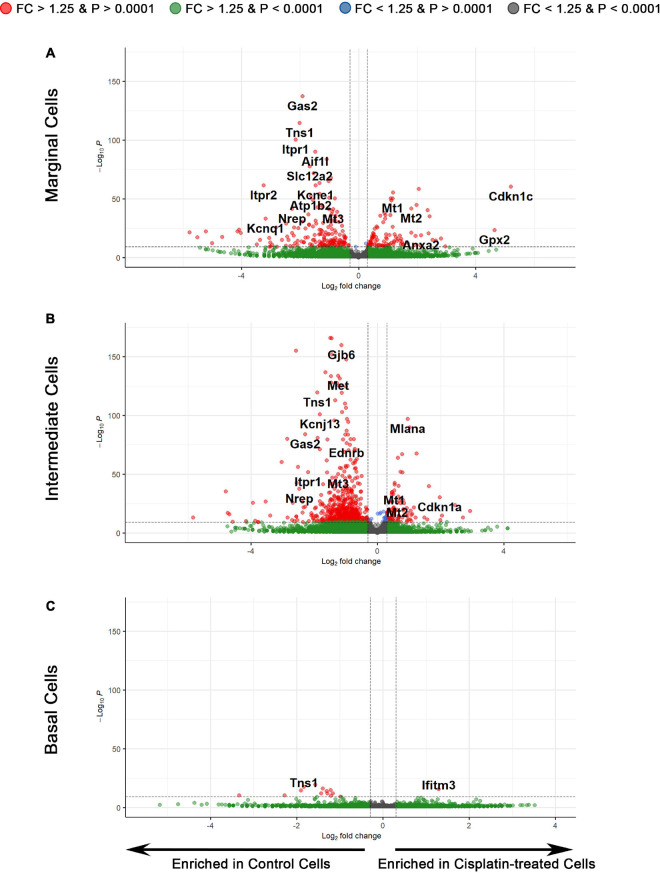
Differential expression analysis reveals the transcriptomic changes in individual cell types of the SV after cisplatin treatment. **(A)** Volcano plot depicting differential gene expression between the scRNA-Seq data of marginal cells of control and cisplatin-treated mice. Genes are represented by circles, with genes enriched in cisplatin-treated SV cells to the right and genes enriched in control SV cells to the left. Positive and negative fold-change thresholds (FC ± 1.25) in log2 scale are represented by the vertical dotted lines. The *p*-value threshold (*p*-value ± 0.0001) in –log10 scale is represented by the horizontal dotted line. Red circles are genes that exceed both fold change and *p*-value threshold criteria. Green circles are genes that exceed the fold change threshold but not the *p*-value threshold. Blue circles are genes that exceed the *p*-value threshold but not the fold change threshold. Gray circles are genes that do not meet either threshold criteria. **(B)** Differential gene expression between the scRNA-Seq data of intermediate cells of control and cisplatin-treated mice. **(C)** Differential gene expression between the scRNA-Seq data of basal cells of control and cisplatin-treated mice.

To examine the transcriptional effects of cisplatin treatment on SV cell types, we performed three separate differential expression (DE) analyses on marginal, intermediate, and basal cells between cisplatin-treated and control SV cells (Figure 5). Volcano plots depict differential gene expression of the marginal (Figure 5A), intermediate (Figure 5B), and basal (Figure 5C) cell types. Volcano plots depict expression of genes, represented by filled circles, enriched in cisplatin-treated SV cells to the right and genes enriched in control SV cells to the left. Fold-change thresholds in log2 scale (fold-change = ± 1.25) correspond to the vertical dotted lines along the horizontal axis and the *p*-value threshold in log_10_ scale (*p*-value = 0.0001) corresponds to the horizontal dotted line along the vertical axis. The higher along the vertical axis, the more significant the *p*-value and the further along in either the negative or positive direction from the dotted line, respectively, the greater the fold-change expression in either the control or cisplatin treated SV cells. Circles in red depict genes that met set threshold criteria (fold-change threshold >1.25, *p* < 0.0001). DE analysis of major SV cell types suggests that cisplatin has a greater effect on SV marginal and intermediate cells as supported by the larger number of differentially expressed genes (Supplementary Table S3). On closer examination, EP-related genes specific for SV marginal cells (*Slc12a2*, *Kcne1*, *Atp1b2*, *Kcnq1*) are enriched in control cells suggesting downregulation of these genes in response to cisplatin in marginal cells (Figure 5A). Furthermore, control SV intermediate cells demonstrate an enrichment for many EP-related genes (*Gjb2*, *Gjb6*, *Kcnj10*, *Met*) suggesting a downregulation of these genes in response to cisplatin (Figure 5B). In contrast to SV marginal and intermediate cells, basal cells demonstrated few differentially expressed genes (Figure 5C). Gene ontology (GO) biological process analysis for genes upregulated in cisplatin-treated cells compared to control cells revealed an enrichment for genes involved in signal recognition particle (SRP)-dependent cotranslational protein targeting to membrane (GO:0006614), ATP synthesis coupled electron transport (GO:0042775), and negative regulation of viral entry into host cell (GO:0046597) in marginal, intermediate, and basal cells, respectively. GO biological process analysis for downregulated genes revealed an enrichment for genes involved in membrane repolarization (GO:0086009), peptidyl-serine phosphorylation (GO:0018105), and mitochondrial ATP synthesis coupled electron transport (GO:0042775) in marginal, intermediate, and basal cells, respectively. GO analysis for both upregulated and downregulated genes suggest that cisplatin has differential effects on cells in the stria vascularis and suggest future areas for potential investigation. Together, these data suggest that SV marginal and intermediate cells demonstrate preferential effects on their transcriptome in response to cisplatin. We provide a list of differentially expressed genes (DEGs) for SV marginal, intermediate, and basal cells between cisplatin-treated and control adult mice (Supplementary Table S3).

Utilizing a larger group of upregulated and downregulated genes, we utilized ddPCR of cisplatin-treated and control whole isolated SV to validate the dataset (Supplementary Figure S2). Upregulated genes that demonstrated consistency between ddPCR and scRNA-Seq included *Cdkn1c*, *Kcnj13*, *Mt1*, and *Mt2* (Supplementary Figure S2A). Despite a trend toward increased expression, the apparent inconsistency for *Anxa2*, *Cdkn1a*, *Gpx2*, *Ifitm*, and *Mlana* between ddPCR and scRNA-Seq could be due to the insensitivity of a whole tissue assay to changes at the single cell level. Downregulated genes including *Aes1*, *Aif1l*, *Gas2*, *Itpr1*, *Itpr2*, *Mt3*, *Nrep*, and *Tns1* demonstrated consistency between ddPCR and scRNA-Seq (Supplementary Figure S2B). Overall, these findings validate downregulation of genes associated with cisplatin treatment.

### Gene Regulatory Network (GRN) Analysis of Cisplatin-Treated SV Using SCENIC Identifies Cell Type-Specific Regulons

Regulons are defined as transcription factors and their motif-enriched downstream target genes. We have previously utilized SCENIC to identify homeostatic cell type-specific regulons in the adult SV (Korrapati et al., 2019). SCENIC analysis along with regulon specificity score (RSS) (Supplementary Figure S3 and Supplementary Table S4) analysis of cisplatin-treated adult SV single cell transcriptomes reveals regulons specific for marginal, intermediate, and basal cells. RSS is a measurement of a regulon’s degree of specificity to a cell type within a single cell data set. Fourteen, twelve, and nine cell-type specific regulons were identified corresponding to marginal, intermediate, and basal cells, respectively. Of these regulons, eight marginal cell regulons, four intermediate cell regulons, and five basal cell regulons were unique to the cisplatin-treated SV. Due to the apparent preferential effect on the transcriptional profiles of marginal and intermediate cells, we focused our attention on marginal and intermediate cell regulons in cisplatin-treated SV. We selected two regulons identified in the cisplatin-treated scRNA-Seq data as examples for our pipeline of cell-type specific regulatory intervention: Kruppel like factor 10 (*Klf10*) in marginal cells and T-Box transcription factor 2 (*Tbx2*) in intermediate cells. In both cases, their RSS scores are above the threshold for specificity for their respective cell types (Supplementary Figure S3A for the Klf10 regulon and Supplementary Figure S3B for the Tbx2 regulon), the transcription factor for each regulon is differentially expressed between control and cisplatin-treated cochlea, and there are multiple downstream targets with FDA approved drugs that either promote or inhibit expression. We utilized ddPCR to quantify the expression of *Klf10* and select downstream targets, including *Atp1b2*, *Tspan1*, and *Cldn3* (Supplementary Figure S4A), as well as the expression of *Tbx2* and select downstream targets, including *Kcnn4*, *Ndufb2*, and *Tubb5* (Supplementary Figure S4B) in cisplatin-treated SV to provide supportive evidence for the existence of these regulons. These analyses allowed us to identify cell type-specific regulons that are present in the cisplatin-treated adult SV.

### Regulon Analyses Identified Potential Therapeutic Gene Targets in Marginal and Intermediate Cells for Cisplatin-Induced Ototoxicity

Given that SV marginal and intermediate cells appeared to be preferentially affected by cisplatin treatment compared to SV basal cells, we focused on selected regulons for SV marginal and intermediate cells based on high RSS scores and presence of EP-related genes. These regulons and their downstream targets serve as examples of potential cell type-specific therapeutic targets. SCENIC analysis identifies the Kruppel like factor 10 (*Klf10*) regulon as a regulon that is present specifically in marginal cells of cisplatin-treated SV. SCENIC analysis did not show presence of the *Klf10* regulon in the control RNA-Seq dataset. *Klf10* has been broadly implicated in epithelial to mesenchymal transition (EMT) as well as TGFβ signaling and is thought to function as a tumor suppressor gene (Venkov et al., 2011; Memon and Lee, 2018). Differential expression (DE) analysis of scRNA-Seq data suggests that transcripts for *Klf10*, Claudin 3 (*Cldn3*), and Tetraspanin 1 (*Tspan1*) are enriched in cisplatin-treated marginal cells. Conversely, DE analysis suggests that transcripts for activated leukocyte cell adhesion molecule (*Alcam*), ATPase Na+/K+ transporting subunit beta 2 (*Atp1b2*), Carbonic anhydrase 12 (*Ca12*), Glutamyl aminopeptidase (*Enpep*), Protein-arginine deiminase type-2 (*Padi2*), and Secreted phosphoprotein 1 (*Spp1*) are downregulated in cisplatin-treated marginal cells (Supplementary Table S3).

In intermediate cells, SCENIC analysis identified that the T-Box transcription factor 2 (*Tbx2*) regulon is present in cisplatin-treated SV. *Tbx2* has been broadly implicated in cell proliferation, induction of EMT, and senescence bypass (Wang et al., 2012; Decaesteker et al., 2018; Crawford et al., 2019). Based on DE analysis, regulon-specific transcripts that were enriched in cisplatin-treated intermediate cells include NADH dehydrogenase 1 beta subcomplex subunit 2 (*Ndufb2*), and Tubulin beta class I (*Tubb5*). Tbx2 regulon-specific transcripts that were downregulated in response to cisplatin include *Tbx2*, Tyrosine-protein kinase (*Abl1*), ATPase Na+/K+ transporting subunit beta 1 (*Atp1b1*), Endothelin receptor type B (*Ednrb*), Hepatocyte growth factor receptor (*Met*), and cAMP-specific 3’,5’-cyclic phosphodiesterase 4B (*Pde4b*) (Supplementary Table S3). Altogether, these analyses suggest that EMT and cell proliferation processes may play a role in the response to cisplatin in the SV marginal and intermediate cells and identify possible druggable gene targets to modulate the response to cisplatin.

## Discussion

This study utilized a mouse model of cisplatin ototoxicity in which a single dose of cisplatin was used to reduce the EP. Our data using this model reveals cell-type specific transcriptional responses in the EP-generating cells of the stria vascularis in response to cisplatin. We demonstrate that SV marginal and intermediate cells are preferentially affected by cisplatin exposure, which is consistent with the role of these cells in generating the EP. Finally, we also demonstrate a pipeline for identifying druggable targets and repurposable drugs using a model of SV dysfunction, which might identify candidates for intervening in cisplatin-related and strial-related hearing loss.

### Single Cell Transcriptional Profiling of Cisplatin-Treated SV Identifies Potential Novel Therapeutic Gene Targets to Mitigate Ototoxicity

We suggest that genes involved in SV function that are downregulated in response to cisplatin may be reasonable targets for augmentation to mitigate the effects of cisplatin on the SV. Therapeutic gene targets that are downregulated in response to cisplatin include *Alcam*, *Atp1b2*, *Spp1*, and *Car12*. Reduced *Alcam* expression has been previously associated with disruption of the blood-brain barrier and its expression may be implicated in blood-labyrinth barrier function (Lécuyer et al., 2017). *Atp1b2*, *Spp1*, and *Car12* are known to be expressed in different cell types in the stria vascularis and lateral wall, notably marginal cells (Lopez et al., 1995; Wangemann, 2002; Wu et al., 2013). *Atp1b2* and *Car12* have been previously implicated in EP generation (Ikeda et al., 1987; Wangemann, 2002). ddPCR validate downregulation of Atp1b2, a gene critical to EP generation, is downregulated in SV marginal cells in response to cisplatin (Figure 3). On the other hand, *Spp1*, or secreted phosphoprotein 1, has not been implicated previously in EP generation. Schmitt and colleagues examined cochlear effects of loss of *Spp1* in response to cisplatin and found no change in *Spp1* expression by immunohistochemistry and no difference in auditory brainstem responses between *Spp1* wild-type and *Spp1* null mice 72 h after cisplatin treatment, concluding that *Spp1* did not appear to mitigate cisplatin ototoxicity (Schmitt and Rubel, 2013). Our scRNA-Seq data demonstrates decreased expression of *Spp1* in marginal cells in response to cisplatin (Supplementary Table S3). Reduced expression of the SPP1 protein after cisplatin treatment was validated using immunofluorescence intensity analysis (Supplementary Figure S5). Properties of *Spp1* include cell death inhibition, which presents a rationale for examining whether augmentation of *Spp1* levels in marginal cells could mitigate cisplatin-induced ototoxicity (Schmitt and Rubel, 2013). Thus, augmenting expression of genes downregulated in response to cisplatin in cell types responsible for EP generation may be an avenue for pursuing otoprotection.

Conversely, we suggest that certain genes that are upregulated in the presence of cisplatin may be reasonable targets for inhibition to mitigate the effects of cisplatin on the SV. Novel therapeutic gene targets that are upregulated in response to cisplatin include *Klf10*, *Cldn3* encoding claudin 3, and Tspan1 encoding Tetraspanin 1, which are part of the *Klf10* regulon. While not previously characterized in the inner ear, *Klf10* overexpression has been implicated in renal podocyte dysfunction in diabetic nephropathy with suppression of *Klf10* shown to reduce diabetes-induced proteinuria and kidney injury in mice (Lin C. et al., 2019). Despite similarities between the kidney and the stria vascularis (Spector and Carr, 1979; Walker et al., 1990; Beitz et al., 1999; Yamamoto et al., 2009), the known interacting partners of *Klf10* in the kidney (Lin C. et al., 2019), including *Kdm6a*, nephrin (*Npsh1*), and methyltransferase *Dnmt1*, are not differentially expressed in the stria vascularis (see Supplementary Table S3). Our *Klf10* regulon analysis identifies *Tspan1* as a potential downstream target that is differentially regulated in marginal cells in response to cisplatin (Supplementary Figure S6A). Tetraspanins are thought to act as molecular scaffolds for adhesion, signaling, and adaptor proteins (Termini and Gillette, 2017). While the role of *Tspan1* as well as the larger family of tetraspanins remains largely undefined in the inner ear, increased expression of *Tspan1* has been observed in a wide variety of cancers including gastric, colon, pancreatic, liver and esophageal cancer and has been implicated in cancer cell migration and invasion (Zhang et al., 2019). Whether these upregulated genes act as compensatory versus detrimental adaptations in response to cisplatin remains to be determined. If detrimental, these gene targets may be candidates for inhibition to mitigate ototoxicity.

### Identification of Novel Potential Therapeutic Targets to Identify Drug Candidates That Could Be Repurposed to Reduce Strial Damage

In order to enhance the robustness of our drug repurposing analyses, we utilized two druggable genome databases, Pharos (Nguyen et al., 2017) and DrugBank (Wishart et al., 2018). Utilizing these databases, druggability of differentially expressed genes in the *Tbx2* regulon revealed potential therapeutic gene targets in intermediate cells in the form of target genes that were druggable by FDA approved drugs (Supplementary Figure S6B). Pharos has a specific focus on drugs that target G-protein coupled receptors, protein kinases, and ion channels (Nguyen et al., 2017), and has been utilized previously by our group to identify potential druggable gene targets (Korrapati et al., 2019). Within Pharos, target genes that are druggable by at least one FDA approved drug are designated as Tclin target genes. Gene targets with FDA approved drugs in DrugBank are searchable and are shown along with known and unknown interactions of FDA-approved drugs. As a result of our druggable targets analysis of differentially expressed genes in the intermediate cell-specific *Tbx2* regulon, we identify metformin as a potential repurposable drug candidate targeting *Ndufb2* (Chen et al., 2019; Muri et al., 2019) to test for potential to mitigate cisplatin-induced ototoxicity as an example of this repurposing drug candidate approach. *Ndufb2* is part of the mitochondrial respiratory chain complex I assembly and has been implicated previously in bacterial sepsis (Li et al., 2017) and Alzheimer’s disease (Manners et al., 2018), suggesting that it may play a role in inflammation. Metformin, a well-known medication used to treat type 2 diabetes, has both anti-inflammatory (Martin-Montalvo et al., 2013; Kelly et al., 2015) and antitumor (Coyle et al., 2016; Hosoya et al., 2017; Zi et al., 2018; Feng et al., 2020) properties with several clinical trials currently underway to study its potential benefit in the setting of cancer (Chae et al., 2016). The antitumor properties of metformin are thought to be potentially mediated through the MTOR signaling pathway (Zi et al., 2018). *Ndufb2* encodes the B2 subunit of NADH:ubiquinone oxidoreductase. Anti-inflammatory properties of metformin may in part be mediated by its ability to limit the production of reactive oxygen species by NADH:Ubiquinone Oxidoreductase, which may limit induction of pro-inflammatory interleukin-1β and boost anti-inflammatory interleukin-10 (Kelly et al., 2015). Recently, metformin has been found to have some beneficial properties as it relates to hearing protection in preclinical and clinical studies against a variety of insults to the inner ear including radiation, noise, and cisplatin, as well as in the recovery of hearing in the setting of sudden sensorineural hearing loss (Chang et al., 2014; Hosoya et al., 2017; Kesici et al., 2018; Chen et al., 2019; Muri et al., 2019; Feng et al., 2020). These observations suggest the possibility that metformin may have broader therapeutic applications as it relates to hearing.

### Understanding the Mechanisms by Which Cisplatin Alters Strial Function Prior to Hair Cell Loss May Inform Strategies to Prevent Hair Cell Loss

In the model utilized in this study, we examined the acute changes that occur in the cochlea in the presence of a reduced EP. Outer hair cells are more susceptible to cisplatin-induced death than inner hair cells (Cheng et al., 2005; Rybak et al., 2007; Li et al., 2011; Breglio et al., 2017; Fernandez et al., 2019), as is the case in other models in which reduced EP precedes hair cell death. In genetic models of EP dysfunction (Liu H. et al., 2016; Huebner et al., 2019; Morell et al., 2020) without a known ototoxin, outer hair cell loss occurs in a delayed fashion after the observed reduction in EP despite the appearance of normally developed hair cells. As the SV generates the EP and establishes the environment for proper hair cell functioning, we speculate that hearing loss prior to outer hair cell degeneration in the setting of EP dysfunction may be due to the sustained effect of a reduced EP on active cochlear amplification by outer hair cells and that the loss of EP may be involved indirectly in the degeneration of outer hair cells (Anniko and Wróblewski, 1986; Jacob et al., 2011; Lukashkina et al., 2017; Zhao, 2017; Morell et al., 2020). Alternatively, work by Sewell demonstrates that reductions in EP resulting from furosemide administration are accompanied by elevations in in auditory nerve thresholds and suggest the loss of EP may have an effect at the auditory nerve (Sewell, 1984). While acknowledging this as speculative, work by our group and others suggests that alterations in the local extracellular ionic composition of the endolymph surrounding the outer hair cells may have an impact on their function and status (Anniko and Wróblewski, 1986; Coffin et al., 2009; Jacob et al., 2011). Alternatively, alterations in EP may initially impact cochlear function prior to effects on the hair cells by impacting auditory nerve fiber activity. Regardless, understanding how disruption of critical SV functions, including EP generation, are associated with hair cell loss, albeit delayed, may be important in identifying ways to prevent hair cell loss and, ultimately, hearing loss.

### Conclusion

We utilized a single dose of cisplatin as a model of SV disease and EP generation dysfunction prior to the onset of deleterious effects of the drug on hearing and hair cells and then examined the transcriptional changes within specific SV cell types. Druggable target databases were then used to predict drugs that might mitigate or reverse these transcriptional changes. Our data at the RNA and protein levels indicate that cisplatin impairs stria vascularis function. We have identified cell-type specific regulatory pathways involved in the response to cisplatin, and these pathways have been used to suggest existing drugs that may mitigate cisplatin ototoxicity. In this way, we identify transcriptional changes that occur apparently prior to the onset of hearing loss that may be therapeutically targeted to prevent cisplatin-related ototoxicity.

## Materials and Methods

### Animals

Inbred CBA/J male and female mice were purchased from JAX (Stock No. 000656). P30 mice obtained from breeding pairs were used for all experiments including EP measurements, immunohistochemistry, digital droplet qPCR (ddPCR) and single-cell RNA-sequencing (scRNA-Seq).

### Cisplatin and Saline Intraperitoneal Injection

Experimental mice were given an intraperitoneal (IP) injection of 14 mg/kg cisplatin 24 h prior to EP measurement or sacrifice. Control mice were given an equal volume IP injection of saline 24 h prior to EP measurement or sacrifice. All mice were age P30 for these experiments.

### Immunohistochemistry

Methods have been previously described (Korrapati et al., 2019; Morell et al., 2020). Briefly, inner ears from P30 CBA/J mice were dissected and placed in 4% paraformaldehyde (PFA) overnight. Fixed adult mouse inner ears were then decalcified in 0.25 M EDTA for 4–5 days, transferred to 30% sucrose in PBS at 4°C overnight, followed by immersion in half 30% sucrose in PBS and super cryoembedding medium (SCEM) (C-EM001, Section-Lab Co, Ltd.; Hiroshima, Japan), followed by embedding in 100% SCEM for 2 h at room temperature, frozen in a cryomold biopsy square.

For cryosectioning, adhesive film (C-FUF303, Section-Lab Co, Ltd.) was fastened to the cut surface of the sample in order to support the section and sectioned slowly using a CM3050S cryostat microtome (Leica, Vienna, Austria) to obtain 10 μm thickness sections. The adhesive film with sections attached was submerged for 60 s in 100% ethanol, then transferred to distilled water. The adhesive film prevents specimen shrinkage and detachment. This methodology allows for high quality anatomic preservation of the specimen and sectioning at a thickness of 0.5 μm. Sections were mounted with SCMM mounting medium (Section-Lab, Hiroshima, Japan) and imaged using a 1.4 N.A. objective.

Fluorescence immunohistochemistry for known SV cell-type markers was performed as follows. Mid-modiolar sections were washed in PBS then permeabilized and blocked for 2 h at room temperature in PBS with 0.2% Triton X-100 (PBS-T) with 10% fetal bovine serum (A3840001, ThermoFisher Scientific, Waltham, MA, United States). Samples were then incubated in the appropriate primary antibodies in PBS-T with 10% fetal bovine serum, followed by three rinses in PBS-T and labeling with AlexaFluor-conjugated secondary antibodies (1:250, Life Technologies) in PBS-T for 1 h at room temperature. Where indicated, 4,6-diamidino-2-phenylindole (DAPI, 1:10,000, Life Technologies) was included with the secondary antibodies to detect nuclei. Organs were washed in PBS three times and mounted in SlowFade Gold (S36937, Invitrogen, ThermoFisher). Specimens were imaged using a Zeiss LSM710 confocal microscope. Sections were mounted with SCEM tissue embedding medium (C-EM001, Section-Lab Co, Ltd.). Primary antibodies used included rabbit anti-KCNJ10 (RRID:AB_2040120, Alomone Labs, APC-035, polyclonal, dilution 1:200), rabbit anti-CLDN11 (RRID:AB_2533259, Life Technologies, 364500, polyclonal, dilution 1:200), goat anti-SLC12A2 (RRID:AB_2188633, Santa Cruz Biotech, sc-21545, polyclonal, dilution 1:200), and Phalloidin AlexaFluor 647 (RRID: AB_2620155, Invitrogen, A22287, dilution 1:250).

### Endocochlear Potential Measurement

Methods for endocochlear potential (EP) measurement have been described (Wangemann et al., 2004, 2007) and have been previously performed by our group (Imtiaz et al., 2018; Sharlin et al., 2018; Morell et al., 2020). Here, adult CBA/J mice were anesthetized with 2,2,2-tribromoethanol (T4842, Sigma-Aldrich, St. Louis, MO, United States) at a dose of 0.35 mg/g body weight. EP measurements were made using glass microelectrodes inserted into the round window and through the basilar membrane of the first turn of the cochlea. Induction of anoxia, allowing measurement of anoxic-state EP, was accomplished by intramuscular injection of succinylcholine chloride (0.1 μg/g, NDC-0409-6629-02, Pfizer, New York, NY, United States) after establishment of deep anesthesia followed by additional injection of 2,2,2-Tribromoethanol (T4842, Sigma-Aldrich, St. Louis, MO, United States). Anoxic-state EP provides an indicator of the lowest EP and sensory hair cell function. In the presence of functional hair cells, the anoxic-state EP is negative, whereas the EP is zero if the hair cells are not functional. Data were recorded digitally (Digidata 1440A and AxoScope 10; Axon Instruments) and analyzed using Clampfit10 (RRID:SCR_011323, Molecular Devices, San Jose, CA, United States). For EP measurements, control (*n* = 12 mice) and cisplatin-treated (*n* = 10 mice) mice were utilized with each mouse serving as a biological replicate.

### Auditory Brainstem Response and Distortion Product Otoacoustic Emissions

Methods have been previously described (Morell et al., 2020). Briefly, auditory brainstem responses (ABRs) were measured in both ears for P30 control (*n* = 6) and cisplatin-treated (*n* = 6) CBA/J mice. Mice were anesthetized with an intraperitoneal injection of ketamine (56 mg/kg) and dexdomitor (0.375 mg/kg) and placed on a heating pad connected to a temperature controller (TC-2000, World Precision Instruments) inside a sound-attenuated booth (Acoustic Systems). The heating pad and thermometer placed under the mouse body were used to maintain body temperature near 37°C. Recordings were obtained using Tucker-Davis Technologies hardware (RZ6 Processor) and software (BioSigRZ, version 5.7.5). For ABR testing, subdermal needle electrodes (Rhythmlink) were placed at the vertex, under the test ear, and under the contralateral ear (ground). Blackman-gated tone burst stimuli (3 ms, 29.9/s, alternating polarity) were presented to the test ear at 8, 16, 32, and 40 kHz via a closed-field Tucker-Davis Technologies MF-1 speaker. Responses were amplified (20×), filtered (0.3–3 kHz), and digitized (25 kHz) with 512–1024 artifact-free responses per waveform. For each frequency, testing began at 80 dB SPL and decreased in 10 dB steps until the ABR waveform was no longer discernable. Once the response was lost, testing continued in 5 dB steps with a minimum of two waveforms per stimulus level to verify repeatability of ABR waves. ABR thresholds were determined by visual inspection of stacked waveforms for the lowest stimulus level that yielded repeatable waves.

Distortion-product otoacoustic emissions (DPOAEs) were measured in both ears for P30 control (*n* = 6) and cisplatin-treated (*n* = 6) adult CBA/J mice using Tucker-Davis Technologies hardware (RZ6 Multi I/O processor, MF-1 speakers) and software (BioSigRz, version 5.7.5) in conjunction with an Etymotic ER-10B + microphone. Two tones were presented simultaneously at levels of f1 = 65 dB SPL and f2 = 55 dB SPL with the higher frequency tone (f2) set between 4-44.8 kHz (5 points per octave) and f2/f1 = 1.25. Mean noise floors were calculated from levels at six frequencies surrounding the 2f1-f2 DPOAE frequency.

### Stria Vascularis Anatomical Assessment and TUNEL Assay

ImageJ was utilized to calculate the cross-sectional area and thickness of the SV in mid-modiolar sections of both control (*n* = 5 mice) and cisplatin-treated (*n* = 6 mice) CBA/J mice at postnatal day 30 (P30) as previously described (Sharlin et al., 2018; Morell et al., 2020). Briefly, midmodiolar cross-sections of adult mouse cochleae were imaged using a Zeiss LSM710 confocal microscope at 40X magnification at each turn of the cochlea. Using phalloidin staining as an outline for the SV, the images for each apical, medial, and basal turn were measured for changes in total SV cross-sectional area (μm^2^), changes in SV thickness (μm) measured at the thickest point, and changes in the number of nuclei between control and experimental mice.

TUNEL assay (Invitrogen, C10617) was performed per manufacturer provided protocol. Briefly, after washing with PBS, samples were incubated with a 1X Proteinase K in PBS solution for 15 min at room temperature and then washed again in PBS. The samples were then covered in terminal deoxynucleotidyl transferase (TdT) reaction buffer and incubated for 10 min at 37°C. The TdT reaction buffer was removed and replaced with a solution of TdT reaction buffer, EdUTP, and TdT enzyme and incubated for 60 min at 37°C. The samples were rinsed in DI water and washed with a solution of 3% BSA and 0.1% Triton X-100 in PBS for 5 min at room temperature. Samples were then incubated with a solution of Click-iT^TM^ Plus TUNEL Supermix and 10X Click-iT^TM^ Plus TUNEL Reaction buffer additive and incubated for 30 min at 37°C, away from light. The samples were then washed in 3% BSA in PBS for 5 min at room temperature and rinsed in 1X PBS. Samples were then washed with PBST three times and incubated for 1 h at room temperature with Phalloidin AlexaFluor 647 (Invitrogen, A22287, dilution 1:250) and DAPI diluted with 10% Fetal Bovine Serum (FBS) in PBST. Samples were then washed with PBST three times and mounted.

TUNEL-positive nuclei were used as an indicator of apoptosis in cells. The apoptotic rate was calculated as the quotient of TUNEL-positive nuclei over the total number of nuclei labeled with DAPI, multiplied by 100, to calculate a percentage of total apoptotic cells within each turn.

### Immunofluorescence Staining

Control (*n* = 5) and experimental (*n* = 6) adult CBA/J mice were given saline and 14 mg/kg cisplatin IP injections 24 h prior to sacrifice and dissection. To prepare samples for freezing, inner ears were dissected and placed in 4% paraformaldehyde (PFA) overnight. Ears were then placed in 0.25 M ethylenediaminetetraacetic acid (EDTA) in phosphate-buffered saline (PBS) at 4°C overnight. Next, they were placed in 30% sucrose in PBS at 4°C overnight, followed by immersion in half 30% sucrose in PBS and super cryoembedding medium (SCEM, SECTION-LAB Co. Ltd.) for 2 h. Finally, they were placed in full SCEM for 2 h, frozen in a cryomold biopsy square, and sectioned onto slides using a cryostat.

For immunofluorescence staining, dried slides were placed on slide warmer for 15 min and then washed with phosphate-buffered saline with tween 20 (PBST) for 10 min. Samples were blocked with 10% FBS in PBST for 2 h at room temperature. Primary antibodies were diluted in 10% FBS in PBST and slides incubated overnight at 4°C. Samples were washed with PBST three times and incubated for 1 h at room temperature with AlexaFluor-conjugated secondary antibodies and DAPI diluted with 10% Fetal Bovine Serum (FBS) in PBST. Samples were then washed with PBST three times and mounted. Primary antibodies used included goat anti-SLC12A2 (Santa Cruz Biotech, sc-21545, polyclonal, dilution 1:100), rabbit anti-KCNJ10 (Alomone Labs, APC-035, polyclonal, dilution 1:100), rabbit anti-CLDN11 (Life Technologies, 364500, polyclonal, dilution 1:100), and Phalloidin AlexaFluor 647 (Invitrogen, A22287, dilution 1:250).

### Immunofluorescence Intensity Analysis of SV Cell Type-Specific Markers

Immunofluorescence intensity analysis was performed as previously described (Sharlin et al., 2018; Morell et al., 2020). Briefly, imaging was performed using a Zeiss LSM710 confocal microscope at 40X magnification at each turn of the cochlea. After converting images to grayscale, fluorescence intensity quantification was performed in ImageJ by calculating the fluorescence intensity of the outlined region of the SV as previously described (Hartig, 2013). Fluorescence intensity was normalized by comparing the SV fluorescence intensity to that of a corresponding region in the scala media. Measurements for the upper (apical), middle, and lower (basal) turns of the cochlea were obtained. These measurements were obtained for known SV cell types including marginal cells (SLC12A2), intermediate cells (KCNJ10), and basal cells (CLDN11). KCNJ10 fluorescence intensity in the spiral ganglion neurons served as a control for immunofluorescence signal intensity measurements. Spiral ganglion fluorescence intensity was unchanged between cisplatin-treated and untreated mice with no statistically significant difference between fluorescence intensity measurements (data not shown). A two-way ANOVA comparison was used to measure statistical significance between conditions for each antibody.

### Hair Cell Counts

Cochlear whole mounts from P30 control (*n* = 6) and cisplatin-treated (*n* = 6) adult CBA/J mice were immuno-stained with anti-MYO7A antibody (Axxora, LLC) for hair cells and DAPI (Life Technologies) for cell nuclei. Organ of Corti sections were microdissected and mounted on slides. Inner and outer hair cell counts were performed from two 210 μm length regions from two cochlear turns (apical and basal) and the mean hair cell count was determined.

### Digital Droplet qPCR (ddPCR)

Control (*n* = 4) and experimental (*n* = 4) adult (P30) CBA/J mice were given a single IP injection of saline or 14 mg/kg cisplatin, respectively, and sacrificed after 24 h. To prepare samples for ddPCR, inner ears were dissected in PBST over ice and SV tissue carefully removed to avoid including unwanted cell types. RNA was retrieved using the Arcturus PicoPure RNA isolation kit and converted to cDNA. TaqMan probes of 17 genes of interest were designed and ordered from Applied Biosystems. Three 96-well plates were prepared to include samples from four control and four experimental mice in triplicate. PCR was done in a 20 μL total volume of 10 μL Bio-Rad ddPCR Supermix for Probes (No dUTP), 1 μL probe of gene of interest, 1 μL reference gene Actb, 1 μL diluted cDNA, and 7 μL molecular grade water. The ddPCR Droplets were generated using the QX200 AutoDG Droplet Digital. PCR was performed as described in the QX200 ddPCR EvaGreen Supermix instructions. Droplets were read with a QX200 Droplet Reader (BioRad) and analyzed with QuantaSoft software (BioRad). Primer sequences utilized for ddPCR are provided in the supplement (Supplementary Table S2).

### Single Cell RNA-Seq

A group of P30 CBA/J mice (*n* = 6, three female, three male) were given a single IP injection of 14 mg/kg cisplatin and sacrificed after 24 h. SV from these mice were micro-dissected as previously described (Korrapati et al., 2019) and utilized to generate single cell transcriptomes. To prepare samples for scRNA-Seq, inner ears were dissected in Dulbecco’s Modified Eagle Medium (DMEM) over ice and SV tissue carefully removed to avoid including unwanted cell types. SV tissue was then placed in a tube containing 200 μL DMEM. 10 μL trypsin was added to the tube and incubated for 7 min at 37°C. After incubation 150 μL liquid was removed and replaced with 150 μL 10% FBS in DMEM. The tissue was triturated for 2 min to dissociate single cells and then passed through a 20 μm filter. Two μL of supernatant was removed, leaving a 100 μL final volume. The concentration was then measured using a cell counter and the final concentration used to determine the volume to be placed in the well of a 10 × 8-channel microfluidics chip. Within this chip droplets were created that each contained a single cell and single uniquely barcoded bead. The cells were then lysed, and RT performed to convert mRNA to cDNA. Single cell libraries were prepared, and the results sequenced on an Illumina sequencer. The details regarding scRNA-Seq workflow are as described in Zheng et al. (2017). After scRNA-Seq protocol and library prep, the resultant sparse matrix was imported into R and clustered using the Seurat package.

### Seurat Clustering

Following Illumina sequencing of the scRNA-Seq results, three files were created: a list of individually barcoded cells, a list of gene symbols with their Ensembl IDs, and a sparse matrix of the read counts of each gene in each cell. These files were then imported into R and converted to a dgTMatrix; a form suitable for analysis using the Seurat package in R. A total of 2,681 cells were captured in the experiment, with 2,236 cells used in the final analysis following filters to minimize doublets and debris. The median number of genes identified per cell was 1,312, while the median number of reads per cell was 3,669. The clusters created by the Seurat package were identified and labeled using published markers of cell types within the SV and lateral wall of the murine cochlea. The methods behind Seurat clustering and differential expression have been previously described (Satija et al., 2015).

### Comparison of Cisplatin-Treated SV Single-Cells Transcriptomes to Untreated Adult SV Single Cell Transcriptomes

Cisplatin-treated adult SV single-cell transcriptomes were compared to a previously published dataset consisting of unperturbed adult SV single-cell transcriptomes (Korrapati et al., 2019) (GEO Accession ID: GSE136196; see footnote 2). The two datasets were combined using the *MergeSeurat* function and normalized after the merge. Cellular identities were carried over from the individual Seurat clustering as discussed in the previous section. Differential expression analysis was performed using the *FindAllMarkers* function in Seurat (Satija et al., 2015) and the DEsingle package (Miao et al., 2018). DESingle is a differential expression package that utilizes raw reads from single cell experiments and performs its own normalization and batch correction. The results of the differential expression analyses were visualized using the EnhancedVolcano package (Blighe et al., 2018) in R.

### Single-Cell Regulatory Network Inference

Single-cell regulatory network inference (SCENIC) and clustering analysis was performed on the post-Seurat analysis object using the methods described initially by Aibar et al. (2017) and applied to the analysis of single cell transcriptomes of the adult SV (Korrapati et al., 2019). Cisplatin-treated SV cells analyzed in the SCENIC pipeline were given identities based on clustering analysis from Seurat to allow for *post hoc* identification of cell types. Briefly, cell type-specific regulons were identified in cisplatin-treated adult SV, regulon activity was quantified, and a regulon activity matrix was constructed to identify cisplatin-treated cell states. Expression of the genes within these regulons was then compared against the genes demonstrating differential expression between cisplatin-treated and control SV single cell transcriptomes obtained by using DESingle (Miao et al., 2018).

### Identification of Potentially Druggable Gene Targets

To identify druggable targets within this list of differentially expressed cell type-specific regulon genes, Pharos (see footnote 1) was utilized as previously described (Korrapati et al., 2019). Briefly, Pharos is a web interface for the Target Central Resource Database (TCRD) created by the “Illuminating the Druggable Genome” program, which collates known drug-protein interactions, including both FDA-approved drugs and small molecules (Nguyen et al., 2017). Pharos categorizes each protein with a “target developmental level” according to how much is known on its “druggability” (Owens, 2007). Targets are classified according to available knowledge: (1) Tclin, where there is known interaction with FDA approved drugs, (2) Tchem, where genes have known and unknown interactions with ligands, (3) Tbio, where genes have a known function but no known interactions, and (4) Tdark, where there is very little known either about function or interaction. To focus on the most clinically relevant targets, we identified Tclin and Tchem genes that were differentially expressed between control and cisplatin-treated scRNA-Seq datasets that were also present in the cell-type specific regulons identified by the SCENIC program. The goal was to identify possible drug interventions in genes and regulatory networks known to be involved in the maintenance of the EP that were also either up or down regulated in the presence of cisplatin.

### Statistical Analysis

For pairwise comparisons between control and cisplatin-treated CBA/J mice, an unpaired two-tailed Student’s *t*-test was used. Comparisons of multiple assays across the same tissue were corrected for multiple comparisons using a Benjamini–Hochberg correction with a false discovery rate (FDR or *q* value) of 5%. Two-way ANOVA and Sidak’s multiple comparisons test were used for comparisons between control and cisplatin-treated mice involving cochlear turns. All statistical analyses were performed using GraphPad Prism version 6.0 (RRID:SCR_002798 GraphPad) for PC. For measurements of strial thickness, cross-sectional area and fluorescent intensity, all values are means ± standard deviations (SD). Both males and females were tested, and there was no evidence of a significant effect of sex on any measures. Therefore, the data displayed in all graphs are from males and females combined.

## Data Availability Statement

All data generated in these studies have been deposited in the Gene Expression Omnibus (GEO) database (GSE165662) and can be found on GEO at: https://www.ncbi.nlm.nih.gov/geo/query/acc.cgi?acc=GSE165662. Single-cell data have been uploaded into the gene Expression Analysis Resource (gEAR), a website for visualization and comparative analysis of multiomic data, with an emphasis on hearing research (https://umgear.org/p?l=d22c4230). All other data associated with this study are available in the main text or the Supplementary Material.

## Ethics Statement

The animal study was reviewed and approved by Animal Care and Use Committee of the National Institute of Neurological Diseases and Stroke and the National Institute on Deafness and Other Communication Disorders, National Institutes of Health.

## Author Contributions

IT, RO, SK, and KF contributed to isolation of single cells for single cell RNA-Seq (scRNA-Seq). EB and RJM were responsible for sequencing and alignment of scRNA-Seq and snRNA-Seq datasets. IT and MH contributed to scRNA-Seq bioinformatic data analysis. IT and RO were responsible for smFISH and immunohistochemistry. IT, KF, EB, RJM, LLC, and MH contributed to writing and revising the manuscript. All the authors read and approved final manuscript.

## Conflict of Interest

The authors declare that the research was conducted in the absence of any commercial or financial relationships that could be construed as a potential conflict of interest.

## Publisher’s Note

All claims expressed in this article are solely those of the authors and do not necessarily represent those of their affiliated organizations, or those of the publisher, the editors and the reviewers. Any product that may be evaluated in this article, or claim that may be made by its manufacturer, is not guaranteed or endorsed by the publisher.

## Footnotes

1
https://pharos.nih.gov

2
https://umgear.org//index.html?layout_id=b50cae7a

## Supplementary Material

Supplementary Figure 1 A single large dose of cisplatin shows no loss of inner or outer hair cells or elevated DPOAE threshold in either ear after 24 h. **(A)** Inner hair cell counts of control (*n* = 6) and cisplatin-treated (*n* = 6) mice were unchanged in either apical or basal halves of the cochlea between conditions (two-way ANOVA, *p* = 0.6434). **(B)** Outer hair cell counts were unchanged in either apical or basal halves of the cochlea between conditions (two-way ANOVA, *p* = 0.6423). **(C)** Left ear DPOAE levels were unchanged between conditions (paired *t*-test, *p* = 0.1823). **(D)** Right ear DPOAE levels were unchanged between conditions (paired *t*-test, *p* = 0.0911).

Supplementary Figure 2 DDPCR validation of upregulated and downregulated genes in control and cisplatin-treated whole stria vascularis as identified by DESingle. **(A)** Upregulated genes: *Anxa2* (unpaired *t*-test, *p* = 0.1392), *Cdkn1a* (unpaired *t*-test, *p* = 0.0490), *Cdkn1c* (unpaired *t*-test, *p* = 0.0001), *Gpx2* (unpaired *t*-test, *p* = 0.3055), *Ifitm3* (unpaired *t*-test, *p* = 0.0961), *Mlana* (unpaired *t*-test, *p* = 0.9097), *Mt1* (unpaired *t*-test, *p* = 0.0002), *Mt2* (unpaired *t*-test, *p* = 0.0002). **(B)** Downregulated genes: *Aes1* (unpaired *t*-test, *p* = 0.0101), *Aif1l* (unpaired *t*-test, *p* = 0.0017), *Gas2* (unpaired *t*-test, *p* = 0.0005), *Itpr1* (unpaired *t*-test, *p* = 0.0004), *Itpr2* (unpaired *t*-test, *p* = 0.0048), *Mt3* (unpaired *t*-test, *p* = 0.0123), *Nrep* (unpaired *t*-test, *p* = 0.0087), *Tns1* (unpaired *t*-test, *p* = 0.0002).

Supplementary Figure 3 Regulon specificity scores ranked according to their specificity to major cell types of the SV. Heavy dotted line is the mean RSS score and the lighter dotted line is one standard deviation from the mean. Regulons marked in blue meet the standard deviation threshold for specificity. **(A)** Marginal cell regulons. **(B)** Intermediate cell regulons and **(C)** Basal cell regulons.

Supplementary Figure 4 DDPCR measurement of the presence of **(A)**, *Klf10* and **(B)**, *Tbx2* regulons. Transcription factors and downstream targets were measured against housekeeping gene *Actb*.

Supplementary Figure 5 Protein expression of SPP1 is significantly reduced in cisplatin-treated SV as measured by immunofluorescence intensity analysis.

Supplementary Figure 6 Pharos druggable targets analysis combined with DE analysis identifies potential therapeutic gene targets and repurposable drugs for cisplatin-induced ototoxicity. **(A)** Illustration depicting the differentially expressed Tclin and Tchem target genes of the marginal cell specific regulon *Klf10*. Genes are colored according to their Pharos gene family identification and interacting drugs and ligands are colored according to their drug-protein interaction. **(B)** The differentially expressed Tclin and Tchem target genes of the intermediate cell specific regulon *Tbx2*.

Supplementary Table 1 Marker genes of UMAP clusters of the cisplatin-treated and control scRNA-Seq datasets as identified by the Seurat *FindMarkers* function.

Supplementary Table 2 Forward, reverse, and probe primer sequences for ddPCR probes.

Supplementary Table 3 DEsingle results for marginal, intermediate, and basal cells between cisplatin-treated and control scRNA-Seq datasets.

Supplementary Table 4 Gene regulatory networks (regulons) identified in the cisplatin-treated scRNA-Seq dataset with their regulon specificity scores.
