# Supplementary figures and images for: Single-Cell RNA-Seq of Cisplatin-Treated Adult Stria Vascularis Identifies Cell Type-Specific Regulatory Networks and Novel Therapeutic Gene Targets
Source: Front Mol Neurosci. 2021 Sep 9;14:718241. doi: 10.3389/fnmol.2021.718241 (PMC8458580; doi:10.3389/fnmol.2021.718241)

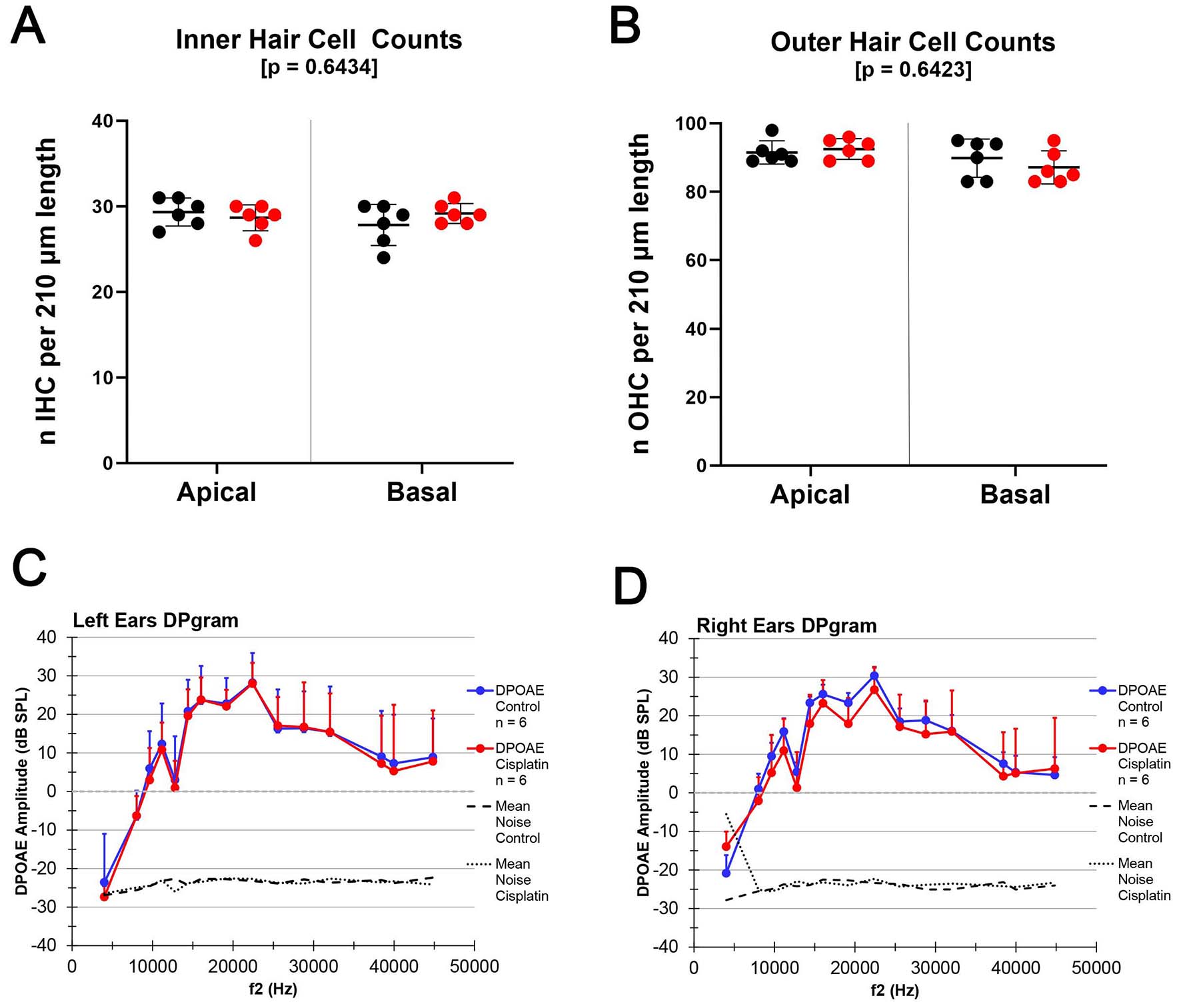

Supplement: Supplementary Figure 1 — A single large dose of cisplatin shows no loss of inner or outer hair cells or elevated DPOAE threshold in either ear after 24 h. (A) Inner hair cell counts of control (n = 6) and cisplatin-treated (n = 6) mice were unchanged in either apical or basal halves of the cochlea between conditions (two-way ANOVA, p = 0.6434). (B) Outer hair cell counts were unchanged in either apical or basal halves of the cochlea between conditions (two-way ANOVA, p = 0.6423). (C) Left ear DPOAE levels were unchanged between conditions (paired t-test, p = 0.1823). (D) Right ear DPOAE levels were unchanged between conditions (paired t-test, p = 0.0911). [file Image_1.JPEG]

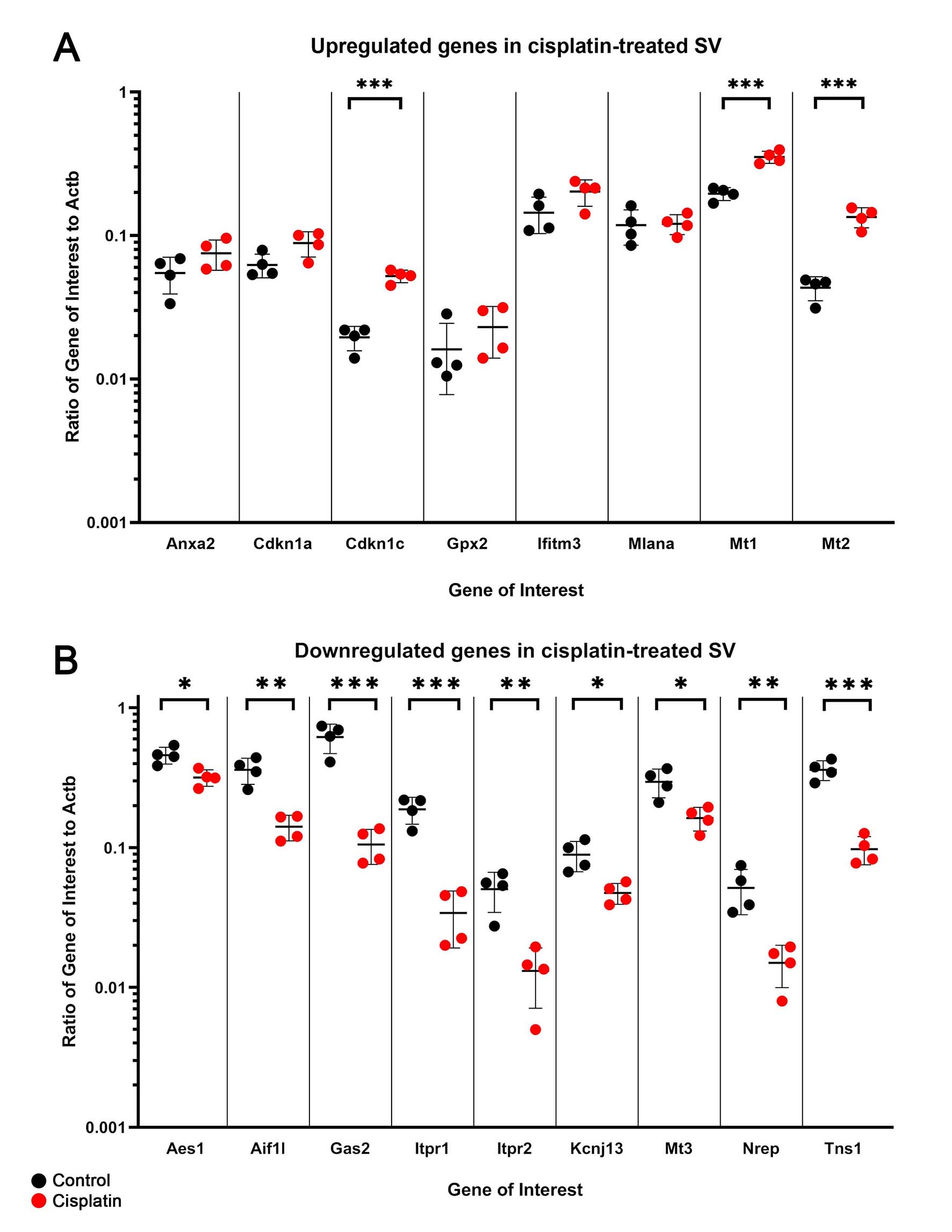

Supplement: Supplementary Figure 2 — DDPCR validation of upregulated and downregulated genes in control and cisplatin-treated whole stria vascularis as identified by DESingle. (A) Upregulated genes: Anxa2 (unpaired t-test, p = 0.1392), Cdkn1a (unpaired t-test, p = 0.0490), Cdkn1c (unpaired t-test, p = 0.0001), Gpx2 (unpaired t-test, p = 0.3055), Ifitm3 (unpaired t-test, p = 0.0961), Mlana (unpaired t-test, p = 0.9097), Mt1 (unpaired t-test, p = 0.0002), Mt2 (unpaired t-test, p = 0.0002). (B) Downregulated genes: Aes1 (unpaired t-test, p = 0.0101), Aif1l (unpaired t-test, p = 0.0017), Gas2 (unpaired t-test, p = 0.0005), Itpr1 (unpaired t-test, p = 0.0004), Itpr2 (unpaired t-test, p = 0.0048), Mt3 (unpaired t-test, p = 0.0123), Nrep (unpaired t-test, p = 0.0087), Tns1 (unpaired t-test, p = 0.0002). [file Image_2.JPEG]

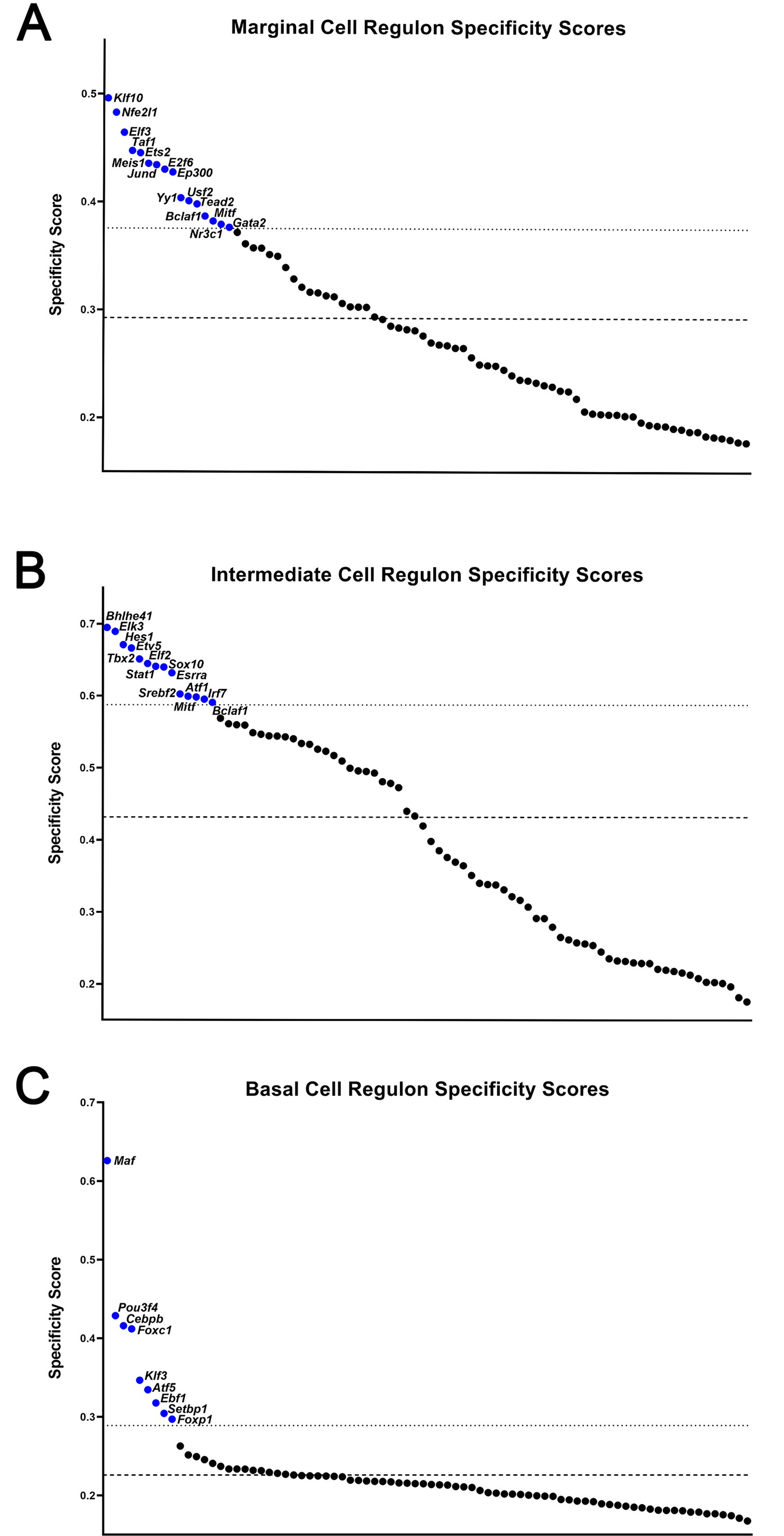

Supplement: Supplementary Figure 3 — Regulon specificity scores ranked according to their specificity to major cell types of the SV. Heavy dotted line is the mean RSS score and the lighter dotted line is one standard deviation from the mean. Regulons marked in blue meet the standard deviation threshold for specificity. (A) Marginal cell regulons. (B) Intermediate cell regulons and (C) Basal cell regulons. [file Image_3.JPEG]

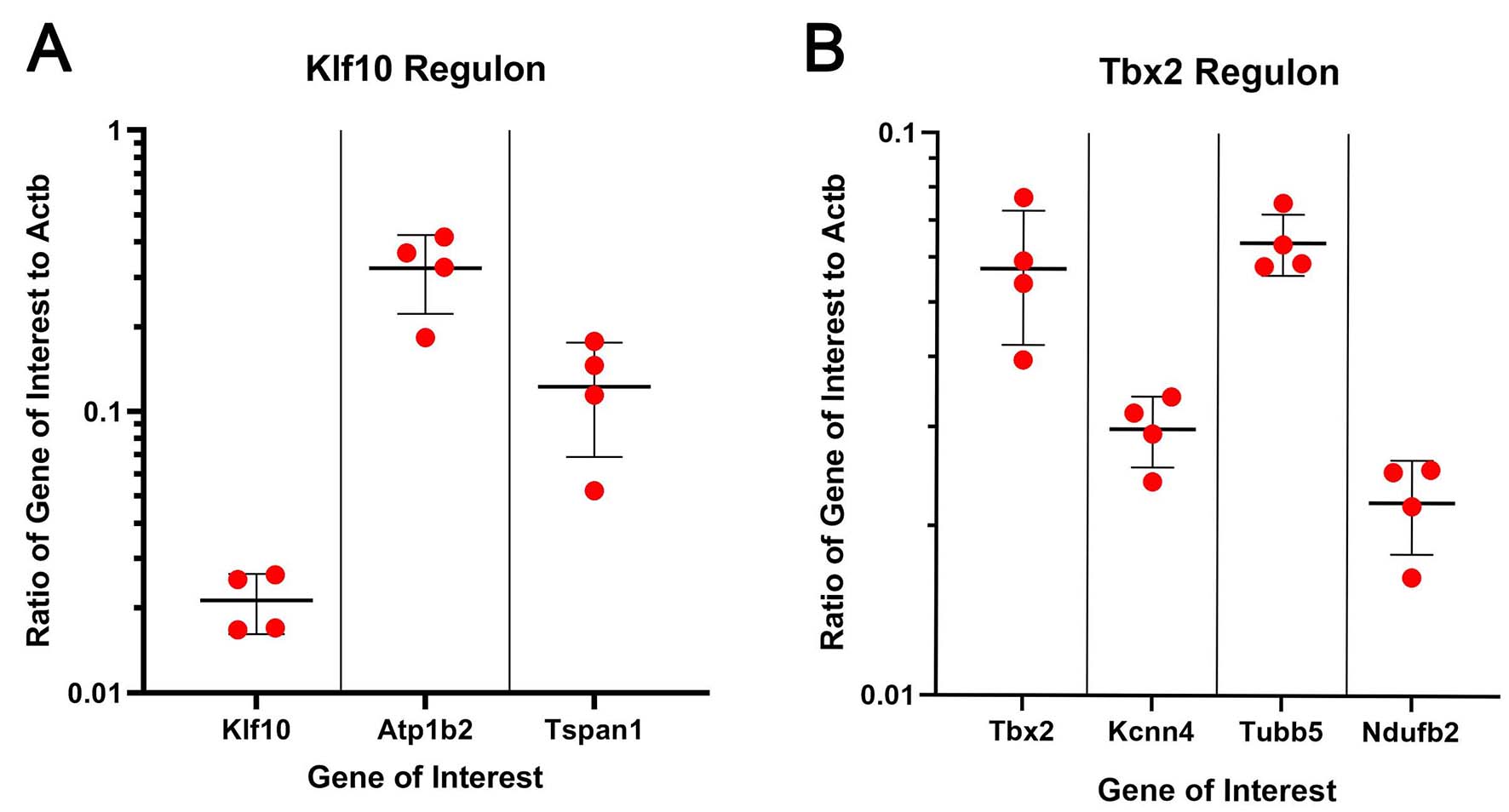

Supplement: Supplementary Figure 4 — DDPCR measurement of the presence of (A), Klf10 and (B), Tbx2 regulons. Transcription factors and downstream targets were measured against housekeeping gene Actb. [file Image_4.JPEG]

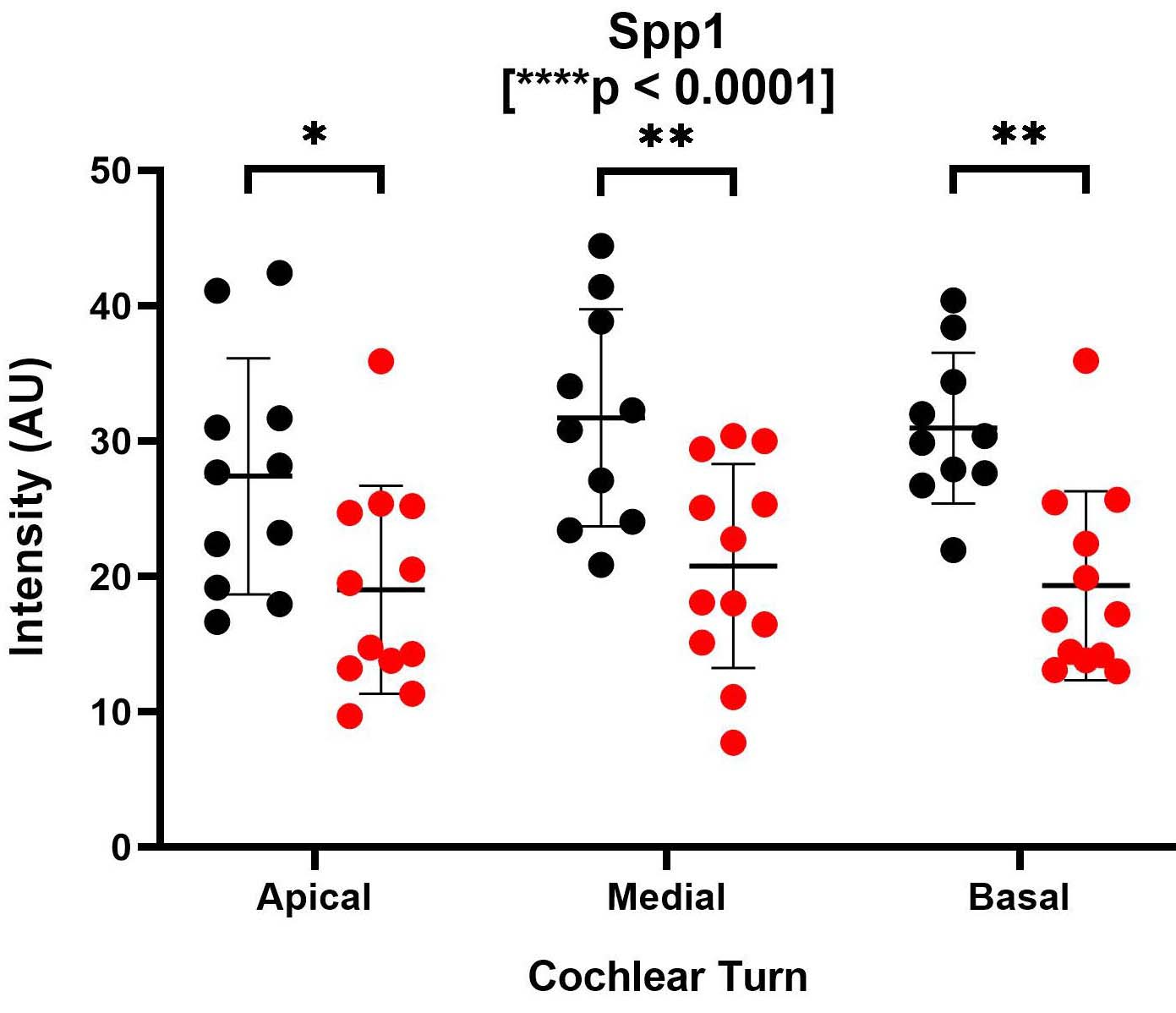

Supplement: Supplementary Figure 5 — Protein expression of SPP1 is significantly reduced in cisplatin-treated SV as measured by immunofluorescence intensity analysis. [file Image_5.JPEG]

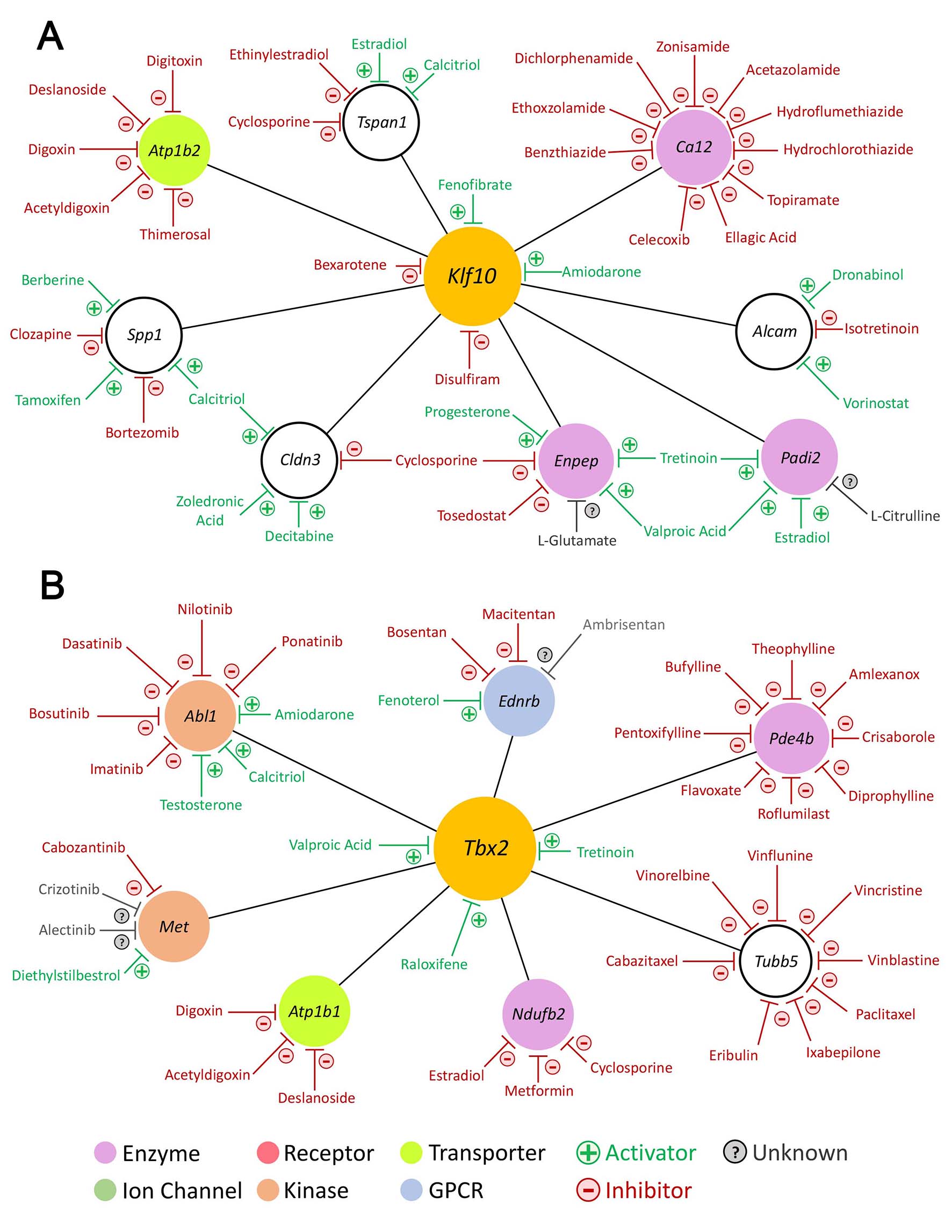

Supplement: Supplementary Figure 6 — Pharos druggable targets analysis combined with DE analysis identifies potential therapeutic gene targets and repurposable drugs for cisplatin-induced ototoxicity. (A) Illustration depicting the differentially expressed Tclin and Tchem target genes of the marginal cell specific regulon Klf10. Genes are colored according to their Pharos gene family identification and interacting drugs and ligands are colored according to their drug-protein interaction. (B) The differentially expressed Tclin and Tchem target genes of the intermediate cell specific regulon Tbx2. [file Image_6.JPEG]
